# Continuous idea contribution in open innovation communities: The role of verbal persuasion from peers

**DOI:** 10.3389/fpsyg.2022.1061415

**Published:** 2022-12-22

**Authors:** Jiacheng Zhang, Guijie Qi, Chunlin Song, Jiali Chen

**Affiliations:** ^1^School of Management, Shandong University, Jinan, China; ^2^School of Management, Shandong Jianzhu University, Jinan, Shandong, China

**Keywords:** open innovation community, continuous contribution, creative self-efficacy, peer feedback, verbal persuasion

## Abstract

Motivating continuous idea contributions from members is especially challenging for open innovation communities (OIC). Scholars have thus explored a range of incentives, among which peer feedback has received much attention. However, existing research on peer feedback tends to focus on the structural features, ignoring the richness of the text. To fill this research gap, this study investigates the influence of feedback language use from peers, including emotional support and constructive feedback, on individuals’ continuous idea contributions, based on the creative self-efficacy theory. The results show that emotional support, especially emotional approval, positively affects members’ continuous contributions, and that the effect is stronger when the provider is of a higher status. However, individualized consideration does not seem to work. In addition, in terms of the effect of constructive feedback, we also find support from cognitive stimulation, while intellectual stimulation exerts an effect only when the provider’s status is high. Overall, these findings extend the current research on peer feedback and offer practical guidelines to the open innovation community.

## Introduction

Acting as an online platform that facilitates collaborative innovation and crowdsourcing, open innovation communities (OICs) attract a large number of innovative users with common interests (Christensen and Karlsson, 2019; Liao et al., 2021). Examples of OICs can be found in diverse domains, such as open source software communities, household sector innovation communities, and firm-hosted crowdsourcing communities for their products or services (Da Silva, 2019; Hober et al., 2021; Li et al., 2022). Such communities help companies to absorb external ideas and optimize the innovation process (Liu et al., 2020), as well as provide community members with a wealth of free innovative ideas to broaden their knowledge (Resch and Kock, 2021). However, these advantages are contingent on the continuous idea contributions from participants. When most participants contribute only a few times or even once, the community’s sustainability may be at risk. Consequently, a key challenge for such online communities is incentivizing participants to contribute continuously.

In order to solve the under-contribution related problems in OICs, previous literature has explored the incentives of participants’ contribution behavior from the psychological, social and functional perspectives. Among them, peer feedback, one of the forms of social interaction, generally referring to other members’ comments on the focal user’s idea, has received much attention from scholars. For example, Chen et al. (2012) claimed that peer feedback has substantial influences on participants’ continuous contributions, and Liao et al. (2021) further verified the effect of peer feedback on idea contributions. However, existing work tended to summarize unstructured peer feedback text with structured proxies, such as the amount, diversity, or timeliness (Chen et al., 2012; Yang and Han, 2019; Chen Q. et al., 2020), which may mask the richness of the text. In addition, although prior studies have differentiated the effect of feedback from the source (e.g., peer feedback or firm feedback; Liao et al., 2021), few have distinguished it in terms of content, such as the role of encouraging and critical feedback, which obviously cannot be viewed equally. Thus, it appears that the textual content of peer feedback and its dimensions are somewhat neglected. Benefiting from the advancement of the automated text-analysis approach, we are now able to study the language use of peer feedback to further explore how it actually affects members’ continuous contributions in OIC settings. Thus, to address the current research gaps, we aim to better understand the promotional effect of peer feedback on continuous idea contributions in OICs by answering the following research question: *How does feedback language use from peers influence members’ continuous idea contributions in OICs?*

To answer this question, we classify peer feedback into two types, namely, emotional support and constructive feedback, based on previous research (Perry-Smith and Mannucci, 2017). Specifically, the former refers to comments that show support and concerns for the idea and the user who proposed the idea, while the latter refers to comments that devote to elaborating the idea any further (Beretta and Søndergaard, 2021; Wang et al., 2021). Drawing on previous studies, we operationalize emotional support by two variables, emotional approval (Wang et al., 2021) and individualized consideration (Becker et al., 2021), representing the language of support and concerns, respectively, to further refine our research. Similarly, we also unfold constructive feedback from two perspectives, namely, cognitive stimulation and intellectual stimulation, representing the language of the cognitive process and questioning tone, respectively (Yang and Han, 2019; Becker et al., 2021). Based on the creative self-efficacy theory, we view peer feedback as verbal persuasion, which is one of the sources of self-efficacy, and explain the promotional effect of peer feedback on participants’ continuous idea contributions. Furthermore, considering the special role of the source of verbal persuasion, we also investigate the moderating effect of peer feedback provider status in the model.

Research is conducted in the household sector innovation setting, which we think is particularly appropriate as a research context. This area is characterized by individuals intrinsically creating ideas and revealing their ideas for free (von Hippel, 2016; Resch and Kock, 2021). Meanwhile, social interaction is frequent and is the main factor driving user engagement, in addition to personal factors. Thus, we crawled a unique panel data set including 30,526 ideas contributed by 15,649 users and a total of 90,813 comments on a popular household sector innovation community. Methodologically, we first extracted linguistic cues from peer feedback utilizing the Linguistic Inquiry and Word Count (LIWC) software, a popular tool for extracting psychological or linguistic constructs from texts. Then, we constructed a panel data regression model to estimate the effect of verbal persuasion from peers on participants’ continuous idea contributions using two-way fixed effects regression.

Our findings indicate that emotional support, especially emotional approval, positively influences members’ continuous contributions and that the effect is stronger when the provider is of a higher status. However, individualized consideration does not seem to work. In addition, we also find support from cognitive stimulation with regard to the effect of constructive feedback. More interestingly, our results show that the role of cognitive stimulation is not affected by the provider’s status, whereas intellectual stimulation exerts an effect only when the provider’s status is high.

Our findings contribute to the literature in terms of both theory and practice. First, we add to the debate about the nature of peer feedback. We answer the call for further investigating the linguistic style of peer feedback and extend prior research on contingency factors (i.e., provider characteristics) of verbal persuasion (Chen et al., 2019b). We provide further insight into how peer feedback offers recipient motivations to continuously generate ideas in OICs based on creative self-efficacy theory. Second, we expand our knowledge of the social factors in household sector innovation that motivate participants to contribute idea continuously. Text analysis techniques enable us to analyze the specific language use of peer feedback and divide it into different dimensions. As a result, our findings suggest that peer feedback does promote continuous user contributions, but it does not apply to all types. Managers are suggested to provide some guidance on the language style of peer feedback in the community, and even consider training automated comment bots accordingly to leverage the promotional effect of specific language use of peer feedback.

## Literature review and theoretical foundation

### Contribution and continuous contribution in the online community

Previous literature in online communities have identified various influencing factors of participants’ contribution behavior, including enjoyment (Wasko and Faraj, 2005), expertise (Zhu et al., 2017), self-image (Chen et al., 2018) and reputation (Liao et al., 2013), etc. In general, members contribute if they expect to receive future benefits (Debaere et al., 2019). However, the antecedents of continuous and initial contribution may be varied, since factors emerging after the first instance may impact the decision to remain engaged (Zhang et al., 2013). Thus, previous research has also investigated the influencing factors of continuous contribution in online communities, and indicates that the factors associated with community members’ continuous contribution intention or behavior can be generally identified from three perspectives: psychological, social and functional (Fang and Zhang, 2019). The psychological dimension mainly refers to individual motivation, which can be further divided into intrinsic (e.g., self-efficacy and learning) and extrinsic (e.g., extrinsic reward) motives (Dong et al., 2020). The social dimension includes reciprocity (Guan et al., 2018), social identity (Dong et al., 2020), and community response or interaction (Chen et al., 2012; Yan and Jian, 2017). In addition, functional dimensions, such as perceived gratifications toward platform attributes (Liu et al., 2020) and guaranteed mechanisms (Jian et al., 2019) can also exert influence on the continuous contribution of participants.

A summary of the literature on influencing factors of participants’ continuous contribution in online communities is presented in Table 1, including the research focus, theoretical basis, data, context, and findings of the study. On the one hand, in terms of research context, while studies related to continuous contribution have produced substantial results, open innovation communities have received less attention. On the other hand, with regard to research focus, existing studies highlight that participants’ continuous engagement is driven by social interactions and the ensuing benefits (Zhang et al., 2013). Specifically, community response or feedback (i.e., comments) to members’ contribution is confirmed to be a key antecedent of their continuous engagement (Chen et al., 2012). Extant research on feedback, however, is mostly limited to the superficial feature of comments (i.e., the amount, diversity or timeliness) on the continuous contribution of individuals, neglecting the role of language use in content of the comment.

**Table 1:** Continuous contribution in online communities.

Study	Research focus	Theoretical basis	Data	Context	Findings	
Chen et al. (2012)	Factors that help explain the participants’ idea quantity, quality, and the duration of active involvement in the community	Reputation and signaling theory	Crawled data	An open innovation community	The level and responsiveness of feedback positively affect participants’ idea contributions and duration of engagement.	
Jin et al. (2015)	Why users contribute to online Q&A communities continuously	Social capital, social exchange, and social cognitive theory	Crawled data	An online Q&A community	Peer recognition, users’ self-introduction, and social learning contribute positively to users’ contribution behavior.	
Chan et al. (2015)	How the characteristics of P2P and P2F user interaction influence their subsequent idea generation	Social identity theory	Crawled data	An idea crowdsourcing community	Both P2P and P2F interactions have significant impacts on users’ subsequent idea contribution. Past ideation participation plays a moderating role in the above relationship.	
Yan and Jian (2017)	How the responses to new entrants’ questions influences their subsequent activities in the community	Social exchange theory	Crawled data	An online Q&A community for programmers	Good answers are detrimental to subsequent contribution of new entrants, but beneficial to future knowledge seeking behavior of new members. Social responses positively affect new entrants’ future community activities.	
Ogink and Dong (2017)	How the peer feedback motivates individuals’ future ideating and commenting	Media usage theory	Crawled data	An open innovation community	Feedback from community members has cognitive, affective and integrative benefits, and they can exert influence on contribution separately and jointly.	
Guan et al. (2018)	Factors that influence users’ continuous knowledge sharing in online Q&A communities	Social capital, social cognitive, and social exchange theory	Crawled data	An online Q&A community	Trust, feedback, social exposure and social norms are positively related to individuals’ continuous contribution behavior.	
Liu et al. (2020)	What are the influencing factors of microblog users’ continuous content contribution behaviors (CCCB)	The uses and gratifications theory, the social influence theory	Survey data	A social media platform	Perceived gratification has a positive impact on users’ CCCB. Meanwhile, social influence has the same effect, as well as positively moderates the above relationship.	
Fang and Zhang (2019)	Factors that promote continuous contributions from members in the online Q&A setting.	Planned behavior theory		Survey data	An online Q&A community	Community commitment, joint vision and common language positively impact users’ attitudes to continued participation. Network externalities can exert effect on participants’ perceived usefulness. The impact on lurkers and answerers have some difference.
Chen et al. (2019a)	Impact of voting and commenting mechanisms on users’ subsequent knowledge contributions to communities	Self-determination theory		Crawled data	A Q&A site	Positive (negative) votes promote (inhibit) users’ sustained contributions. In addition, comments play a moderating role between voting and users’ knowledge contribution.
Wang et al. (2020)	Factors influencing individuals’ sustained participation in crowdsourcing competitions	The social cognitive theory		Crawled data	An online crowd sourcing contest platform	Individual sustained participation is influenced by tenure, prior performance, price amount, number of competitors, and duration of competition
He et al. (2020)	Influence of employees’ psychological needs and motivation on their continuous knowledge contributions	The use and gratifications theory		Crawled data	An enterprise social media platform	Personal and social integration needs, as well as emotional needs are found to promote employees to continuously publish knowledge sharing.
Dong et al. (2020)	How the effect of various motives on continuous contributions are moderated by the status	The status theory of collective action (STOCA)		Crawled data	A third-party consumer review site	Status diminishes the effect of virtual rewards and peer recognition on sustained contributions, but strengthens the impact of opinion leaders on sustained contributions.
Wang et al. (2021)	Impact of various motives on participation behaviors, which further influence continued generating intention	Use and Gratifications (U&G) theory		Survey data	A product-experience-shared community	Different participation behaviors are significantly correlated with utilitarian, recreational, cultural and/or social motivations. Users’ satisfaction with experience significantly affects their continued generating intention.
Zhang et al. (2021)	Factors that influence the contribution of members in social knowledge communities and how they are influenced	Self-efficacy theory and theory of reciprocity		Survey data	Online Learning Group	I-intention and we-intention factors are positively related to users’ continuously knowledge sharing and self-efficacy plays a moderating role in the relationship.

Related research indicates that if online communities are to sustain and grow their operations, attention must be paid not only to factors, such as social networks formed through communication mechanisms (i.e., commenting on others’ contributions) but also to how the communication content itself impacts the willingness of members to expend time and effort to contribute (Chen L. et al., 2020). For instance, Piezunka and Dahlander (2019) emphasized that the linguistic style of the feedback mattered in helping communities keep ideators engaged and gather further ideas. In our research context, internal motivation played a primary role in driving users’ contribution in the absence of external rewards (Piezunka and Dahlander, 2019; Resch and Kock, 2021), and social factors (i.e., peer feedback) that emerged after contribution also have an important impact on their subsequent behavior. In addition to the informational value identified by prior works (Chen et al., 2012), peer feedback can also convey recognition, encouragement and stimulation through language, which have the potential to promote contribution. For example, Wang et al. (2021) emphasized the influential role of informational support and emotional support on members’ contributions through quantitative content analysis. With regard to linguistic style, we argue that it should not be limited to the emotional aspect, but it should explore more linguistic characteristics with text-analysis techniques. Thus, considering the existing research gap and practical importance, the current study attempts to explore the impact of various language uses of peer feedback on innovators’ continuous idea contributions.

### Creative self-efficacy theory and verbal persuasion

To examine the effect of language use in peer feedback on participants’ continuous idea contributions in an open innovation community setting, we draw on a theoretical perspective of creative self-efficacy, and investigate the effect of verbal persuasion, one source of self-efficacy, on promoting participants’ continuous contribution.

In the face of creative work, individuals need some internal, sustaining force to propel them to exert effort. Creative self-efficacy seems to provide such driving force as powerful efficacy faith improves the individual’s insistence and effort level when encountering challenging situations (Bandura, 1977; Amabile, 1983). Subsequent studies have pointed out that, among the personal factors affecting creative performance, creative self-efficacy serves as a crucial role (Tierney and Farmer, 2002; Puente-Díaz, 2016). Derived from Bandura (1977)‘s self-efficacy theory and Amabile (1988)‘s research on creativity theory, creative self-efficacy is defined as the belief that an individual has the ability to produce innovative outcomes (Tierney and Farmer, 2002). Accordingly, individuals build self-efficacy beliefs through four principal sources: mastery experiences, vicarious experiences, verbal persuasion, and physiological states (Bandura, 1977). Mastery experiences are the direct outcomes of past performance, providing the leading source of self-efficacy. Vicarious experiences, in contrast, are information obtained from the result of others’ practices. Verbal persuasion is the act of getting a person to believe that he or she is capable of successfully completing their task, usually takes the form of recognition and feedback from peers. Physiological states represent both physiological and emotional feelings of individuals. Previous research has shown that the above four factors influence the development of individuals’ self-efficacy, which in turn affects innovation performance (Liao et al., 2021).

In contrast from prior work on the source of self-efficacy, the current study focuses only on the effects of verbal persuasion. Because we notice that the existing studies tend to focus on the surface of verbal persuasion, empirically testing how the amount of verbal persuasion influences innovation performance, while neglecting its content aspects. Thus, this paper focuses on the content of verbal persuasion itself, investigating the influence of the language use of verbal persuasion on user innovation by means of text analysis.

Due to the effectiveness of verbal persuasion being influenced by the expertise, credibility, and appeal of the encouragement provider (Bang and Reio, 2017), existing related studies have mostly focused on the language styles of leaders and indicate that leaders’ language and word choices influence followers’ perceptions and behaviors. Fan et al. (2014), for example, claimed that specific language styles applied to give encouraging feedback can affect members’ creative performance. Moreover, Becker et al. (2021) highlighted that the language and words that moderators utilize to communicate with community members in a virtual context affect their participation and innovation endeavors. However, in our research context, many ideas are created by members every day. It is not practical for the community moderator to comment on each idea, which means that peer comments are the main source of verbal persuasion. Considering the above practical situation, we decide to explore the effect of verbal persuasion from peer comments on focal users’ continuous contribution, thereby expanding the research scope of verbal persuasion.

## Hypotheses

Motivated by factors, such as enjoyment of sharing, individuals make their initial idea contribution (Chen et al., 2012), unlocking self-centered community interaction (i.e., peer feedback). The ensuing interaction and the language embedded in it largely influence individuals’ subsequent community participation and idea contributions because it can meet their inherent needs. Perry-Smith and Mannucci (2017) asserted that after the idea is generated, it goes through an elaboration phase where the ideator needs support from others in two forms. First, the ideator needs emotional support to reduce uncertainty and increase confidence to push the idea further (Madjar et al., 2002). A sense of relatedness and being noticed under a community context can grow the intrinsic motivation (e.g., self-efficacy) for individuals to continue contributing to the community (Ryan and Deci, 2000). Second, the ideator also needs constructive feedback and suggestions for improving their ideas and solving challenges (Harrison and Rouse, 2015). This form of support helps ideators grow and develop, increasing their ability to create ideas in an informational way. This kind of support is as important as emotional support for promoting the continuous contribution of individuals.

Prior work has demonstrated that individuals’ creativity was impacted by other members in the OIC (Chan et al., 2015). For example, Chen et al. (2012) found that users who received a large number of peer feedback would possibly contribute more ideas. This article attributes such a promoting effect partly to the power of language. As previously mentioned, verbal persuasion usually refers to peer feedback or recognition from others (Liao et al., 2021), including the form of suggestions, instructions, and judgments. Verbal persuasion, if appropriate, has the ability to develop recipients’ self-efficacy (Lin and Flores, 2013), which in turn promotes new idea generation. In our research context, peers can express their recognition or encouragement to ideators and provide feedback in the comments. To refine the dimensions of comment content, this article divides it into emotional support and constructive feedback based on previous research (Perry-Smith and Mannucci, 2017; Beretta and Søndergaard, 2021), discussing the effect of verbal persuasion, and further examining the role of its source.

### The effect of peers’ emotional support language use

Emotional support refers to comments that express support and concerns for the idea and the ideator (Beretta and Søndergaard, 2021; Wang et al., 2021). Peer feedback can convey approval and consideration to individuals through language in the form of emotional support. Consequently, it makes individuals become aware of their full potential and increases their willingness to create ideas (Becker et al., 2021). Specifically, being recognized and approved by peers in the community is the pursuit of every member, and it not only brings individual enjoyment but also boosts their creative self-efficacy to increase innovation. Meanwhile, encouraged by peers’ emotional approval language, individuals might feel free to implement ideas into practice and tend to try novel and different approaches, which means more idea output in the community. In contrast, if they do not receive encouragement or are criticized, individuals may deny their ideas, as well as their own creativity, which ultimately undermines their intrinsic motivation (Shalley and Perry-Smith, 2001).

In addition, research on leadership language indicates that, through approaching members and exhibiting individualized consideration, leaders can psychologically empower and intrinsically motivate them to increase innovation performance (Afsar et al., 2014). Likewise, in our research setting, community peers often show their care and concern to the focal user by commenting directly on their ideas. In turn, users feel a strong sense of community belonging, which has been proven to be influential to maintain individuals’ continued community engagement (Chen et al., 2019b). Emotional support language also enables individuals to develop a proactive commitment to the community, which promotes their willingness to contribute creative ideas (Chen et al., 2012). Furthermore, prior research emphasizes that online community members abide by generalized reciprocity (Deichmann et al., 2021), which means that individuals tend to actively participate and contribute to the community for others’ care and encouragement in return. From the perspective of both the individual and the community, the emotional support language use of peers may have positive effect on the likelihood of members’ continuous idea contributions. Therefore, we propose the following hypothesis:

*H1*: Peers’ emotional support language use is positively related to members’ continuous idea contributions.

### The effect of peers’ constructive feedback language use

In addition to emotional attitudes, the language of peer feedback can also convey informational value in the form of constructive feedback, which can alleviate the negative effects caused by information asymmetry and stimulate members to contribute further (Jiang and Wang, 2020). Constructive feedback refers to comments that devote to elaborating the idea any further (Beretta and Søndergaard, 2021), including sharing related information and knowledge, suggesting possible improved methods, recombining it with similar ideas, questioning individuals’ assumptions, and calling for new solutions (Becker et al., 2021; Beretta and Søndergaard, 2021). Peers’ constructive feedback language can act as cognitive and intellectual stimulation, which pushes individuals to adopt innovative approaches and develop new ideas (Zhou et al., 2012). In our research context, most individuals are intrinsically motivated to experiment and share ideas and designs. Therefore, peers’ constructive feedback can appeal to more individuals and further stimulate individuals’ efforts on innovative tasks.

On the one hand, the comment language that reflects the cognitive process can arouse users’ critical thinking that contributes to idea generation (Yang and Han, 2019). For example, Ogink and Dong (2017)‘s research on peer feedback found that it has cognitive benefits and promotes individuals’ participation behaviors. On the other hand, community peers can stimulate individuals by challenging their views and methods about the idea in the comments. For example, peers might use a questioning or suggestive tone when commenting on members’ ideas (Boies et al., 2015), and perhaps leave the focal user a URL for further learning and reference, which increases the likelihood of individuals coming up with new ideas. In addition, comments can also generate interest from individuals, inducing their proactive interaction behavior (Den Hartog and Belschak, 2012). The individual being commented on also tends to comment more on others due to the influence of the overall community environment. Consequently, the individual increases the diversity of knowledge in the process of communicating with others and improves the ability of problem solving and innovation integration. Overall, we anticipate the constructive feedback language use of peers to play a cognitively and intellectually stimulating role, as well as motivate users to engage in continuous participation and contribution. Therefore, we hypothesize the following:

*H2*: Peers’ constructive feedback language use is positively related to members’ continuous idea contributions.

### The moderating effect of peers’ status

It is reasonable to assume that the effects of verbal persuasion from various sources are different. When a person with high status and extensive experience, referred to as the model, delivers verbal persuasion, the recipient’s self-efficacy beliefs should be reinforced more (Mellor et al., 2006). For this reason, previous studies have spotlight on the influence of leaders’ language styles on employee behavior. In any online community, there is a certain group of users who are the main source of information and influence, widely recognized by members, and known as opinion leaders (Wang et al., 2020). It is inspiring to be noticed and guided by them. The above scenario also applies to the innovation community. Because the effectiveness of verbal persuasion varies between providers with different levels of expertise and appeal (Bang and Reio, 2017), we propose that emotional support and constructive feedback provided by peers with high status (i.e., opinion leaders) have a stronger effect on the focal user. We therefore hypothesize that:

*H3a*: Status positively moderates the influence of peers’ emotional support on members’ continuous idea contributions (i.e., the impact of emotional support language use on continuous idea contributions is stronger for high-status peers than for low-status peers).

*H3b*: Status positively moderates the influence of peers’ constructive feedback on members’ continuous idea contributions (i.e., the impact of constructive feedback language use on continuous idea contributions is stronger for high-status peers than for low-status peers).

**Figure 1:**
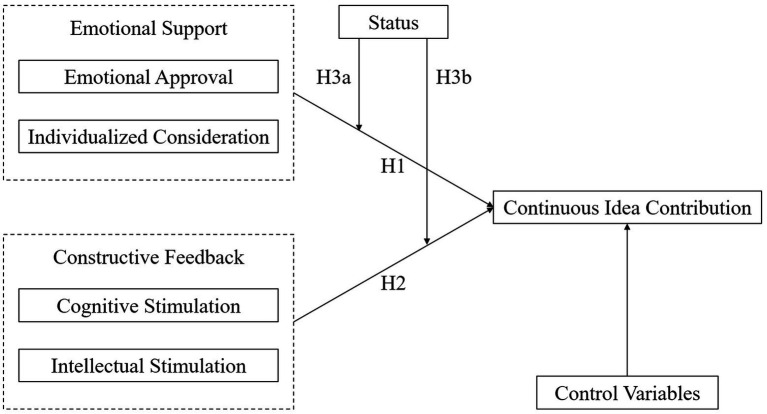
The conceptual model.

In summary, the conceptual model of this study is presented in Figure 1.

## Methodology

### Context and sample

We decide to conduct our empirical study in the context of household sector innovation. Household sector innovation is defined as the ideation and development of new products or modifying existing ones by individuals, revealed freely in their spare time (von Hippel, 2016; Claussen and Halbinger, 2021). We perceive this to be a well-suited research setting to conduct our empirical research for the following reasons. First, household sector innovation is currently trendy and prevalent, representing an increasing part of the overall innovation space (De Jong et al., 2021). It involves innovations in a variety of areas, such as sports equipment, software, and even medical applications (Resch and Kock, 2021). Household sector innovation, thus, holds considerable potential to improve social welfare (Gambardella et al., 2016). Second, household sector innovations are self-rewarded and driven by intrinsic motivation; individuals derive auxiliary benefits (e.g., helping out others, enjoyment, learning new skills) from the process of innovation, and there are no corporate directions interfering. Third, individuals not only innovate solutions for their own use but also frequently provide free access to others for their further use. Thus, both material and immaterial resource, such as protypes and experiences, are exchanged frequently, leading to intense social interaction and peer feedback (Browder et al., 2019). The large amount of data makes it possible to empirically investigate our research questions and produce reliable results.

Consequently, we obtain our dataset from one of the most welcoming household sector innovation communities, which is an open web environment where people can explore, document and share creations. The community claims to be the largest online DIY community in the world, providing free access for registered members to release their own ideas or designs, as well as favor and leave comments on other members’ innovations. Thus, it is widely accepted by makers and household sector innovators.

Utilizing a web crawler, we first collected the available data about all ideas generated by community users over an 18-month period, from May 2020 to October 2021. The data of ideas consists of the title, ideator, release time, category, number of views and favors, as well as a description and production steps of the idea. For each idea, we further record the link to the ideator’s community profile, obtaining their demographic data (e.g., location and joined date) and participation data (e.g., discussion and idea contributions). Meanwhile, we examine all the comment data, which include the commenter, comment text, the time when the comment was posted and the idea to which the comment belongs. Therefore, the dataset includes rich, time-stamped longitudinal data for all ideas and comments of an innovator during a certain period. The entire process generates a total of 30,526 ideas contributed by 15,649 users and a total of 90,813 comments.

### Measurement

To construct the linguistic style variables representing the dimensions of verbal persuasion, we utilize the Linguistic Inquiry and Word Count (LIWC) (Zhou et al., 2012), an automated text analysis tool that supports analyses of how members’ comments are written (Debaere et al., 2019). Based on the pre-categorized dictionary, this approach assesses each comment and assigns a final score according to the percentage of words that belong to a certain word category. Strong evidence indicates that the extracted LIWC variables show good agreement with the ratings of human coders (Zhou et al., 2012; Ludwig et al., 2014), and their validity and reliability have been previously confirmed. Thus, LIWC has been increasingly applied by information systems scholars for text analysis, more specifically, to extract linguistic and psychological constructs from text (e.g., Chen L. et al., 2020; Peng et al., 2020; Park et al., 2021). Consistent with prior research on linguistic styles, we rely on several word categories as input for our research model, including *posemo*, *you*, *cogpro*, and *excl*.

#### Dependent variable

Community members’ continuous idea contribution is the dependent variable in this study. In line with the definition provided by Chen et al. (2019a), we use a count measure of ideas to assess the continuous contribution of each member. To be more precise, we define $Idea_{i,t}$ as the number of ideas submitted by member *i* in period *t* (one period consists of 4 weeks).

#### Independent variables

To operationalize the dimensions of verbal persuasion, we consider direct interactions of the peers with the focal member. Specifically, utilizing LIWC, we extract linguistic features from the peer’s comments on the ideas of the focal member. Then, we use them to construct the measurement of emotional support and constructive feedback language use.

First, we measure emotional support as emotional approval and individualized consideration. Emotional approval is an important feature of expressing recognition and acceptance, which is measured by the emotional score in prior work (Wang et al., 2021). The *posemo* category in LIWC calculates the percentage of positive words (e.g., great, nice, excellent) in a passage. Thus, we apply the *posemo* LIWC category to determine the emotional approval that peers use in direct communication with the focal member. In addition, considering that concerns, such as caring, understanding, and empathy are also important aspects of emotional support (Liang and Turban, 2011), we add an individualized consideration variable (Becker et al., 2021) into the research. Accordingly, the language containing second-person pronouns inherently emphasizes the receiver and their needs, thus demonstrating the consideration from the provider, which can influence the participation behavior of the receivers (Cruz et al., 2017). Therefore, we utilize the *you* category in LIWC to calculate the percentage of second-person pronouns in peers’ comments.

Second, because peer constructive feedback involves not only the cognitive stimulation process, such as perception and suggestions for focal ideas and connection with other ideas but also the intellectual stimulation process, such as questioning individuals’ assumptions and challenging the status quo (Beretta and Søndergaard, 2021). We apply cognitive stimulation-and intellectual stimulation-related words that peers use in comments to operationalize constructive feedback. For the former, we use the *cogproc* category value in LIWC (e.g., should, could, because, think), which is the percentage of words in peers’ comments representing cognitive processes. For the latter, we utilize the *excl* category value in LIWC (e.g., but, without, however), which has been used to reflect the questioning and challenging tone (Becker et al., 2021).

Considering that a focal member posts a certain number of ideas in period t and each idea also receives a certain number of comments from peers, we use the method from Wang et al. (2021) to calculate the value for the above variables. As shown in Formula (1), $N_{i,t-1}$ is the number of ideas up to time period (*t*–1) of focal user *i*. For idea *j* of user *i*, $M_{ij,t-1}$ is the comment number of user i’s idea j up to time period (*t*–1). $LIWC_{i,t-1}$ is the net LIWC score toward the certain category of comment k on user i’s idea j as follows:


(1) $$LIWC_{i,t-1}=\left\{\overset{N_{i,t-1}}{\underset{j=1}{\sum }}\left[\left(\overset{M_{ij,t-1}}{\underset{k=1}{\sum }}LIWC_{ijk}\right)÷M_{ij,t-1}\right]\right\}÷N_{i,t-1}$$

#### The moderating variable

Selecting opinion leaders and displaying them on the community homepage is a popular method for OICs to motivate members to engage in contribution activities. In our research setting, members are aware of the community’s popular users, as the community promotes them on the homepage by showing their profile photos, homepage links and real names. On average, these popular users post more than 80 ideas, receive more than 8,000,000 views, and have a large number of followers. Therefore, we consider this group of users to be the opinion leaders of the community and use a dummy variable to indicate whether users receive comments from any opinion leaders in a given period.

#### Control variables

We control for multiple variables that may have an impact on members’ continuous idea contribution. In terms of individual features, we control variables, including the number of peer comments (Chen et al., 2012), the number of ideas released by the focal ideator before (Chan et al., 2015), the tenure in the online innovation community and its squared term (Dong et al., 2020). We also control various other linguistic style variables that have been confirmed to possess the potential to influence user behavior, including average words count that peers use in comments, words per sentence, words more than six letters and negate words (Cohn et al., 2004; Tausczik and Pennebaker, 2010; Perry-Smith and Mannucci, 2017; Becker et al., 2021). In addition, utilizing fixed effects panel regression models, we also control for unobserved individual characteristics, such as gender, location and other personality traits (Chen et al., 2019b).

Table 2 lists and describes all model variables.

**Table 2:** Variables description.

**Variable**	**Description**
***Dependent variable***	
*Idea_it_*	The number of ideas submitted by user *i* in period *t*.
***Independent variables***	
*EmoApp_it_*	The average score of *posemo* category calculated by LIWC in the comments received by user *i* in period *t*. Range = [0, 100].
*IndCons_it_*	The average score of *you* category calculated by LIWC in the comments received by user *i* in period *t*. Range = [0, 100].
*CogStim_it_*	The average score of *cogproc* category calculated by LIWC in the comments received by user *i* in period *t*. Range = [0, 100].
*IntStim_it_*	The average score of *excl* category calculated by LIWC in the comments received by user *i* in period *t*. Range = [0, 100].
***Moderator***	
*Status_it_*	A dummy variable that equals 1 if user i receive comments from any opinion leaders in a period t and 0 otherwise.
***Control variables***	
*ComRec_it_*	The number of comments received by user *i* in period *t*.
*IdeaPos_it_*	The total number of ideas posted by user *i* up to period *t*.
*Tenure_it_*	The number of months user i has joined the OIC up to period t
*WC_it_*	The average words count of comments received by user *i* in period *t*.
*WPS_it_*	The average number of words per sentence in comments received by user *i* in period *t*.
*Sixltr_it_*	The average score of words more than six letters in comments received by user *i* in period *t*. Range = [0, 100].
*Negate_it_*	The average score of negate words in comments received by user *i* in period *t*. Range = [0, 100].

### Model specification

To verify the research hypothesis proposed in this paper, we build a fixed-effects panel model as follows:


$$\begin{array}{l} Idea_{i,t}=\beta_{1}EmoApp_{i,t-1}+\beta_{2}IndCons_{i,t-1}+\beta_{3}CogStim_{i,t-1}+\\ \beta_{4}IntStim_{i,t-1}+\beta_{5}EmoApp_{i,t-1}\times Status_{i,t-1}+\\ \beta_{6}IndCons_{i,t-1}\times Status_{i,t-1}+\beta_{7}CogStim_{i,t-1}\times \\ Status_{i,t-1}+\beta_{8}IntStim_{i,t-1}\times Status_{i,t-1}+\\ \beta_{9}C_{i,t-1}+\alpha_{i}+month_{t}+\varepsilon_{i,t} \end{array}$$

where $idea_{i,t}$ denotes the idea of user i in month t; $PosEmotions_{i,t-1}$ , $SecPron_{i,t-1}$ , $CogProc_{i,t-1}$ and $Excl_{i,t-1}$ are the average percentages toward the corresponding word category of comments received by user *i* in month *t*–1; $Status_{i,t-1}$ represents whether user *i* received comments from opinion leaders in month *t*–1; $C_{i,t-1}$ represents all the control variables; $\alpha_{i}$ and $month_{t}$ are the individual and time fixed effects, respectively; and $\varepsilon_{i,t}$ represents the error term. Considering that the reverse causality effect occurs when the measurement of independent and dependent variables are conducted simultaneously, for example, members can receive more comments with emotional support when they post ideas, we lag independent and control variables by one period in the estimation equation, to mitigate potential endogeneity issues associated with reverse causality.

## Results

### Descriptive statistics and correlation analysis

Table 3 demonstrates descriptive statistics of all variables, and Table 4 shows the correlations. It indicates a positive correlation between the number of ideas and all other variables. We also examined the VIF values for the presence of multicollinearity. The average value of VIFs is 2.24 and the maximum value is 4.69, indicating that multicollinearity is not a serious issue (Chen et al., 2019a).

**Table 3:** Descriptive statistics.

**Variables**	**Min**	**Max**	**Mean**	**S.D.**
*Idea_it_*	0	21	0.108	0.458
*EmoApp_it_*	0	99	3.974	18.286
*IndCons_it_*	0	50	0.122	1.065
*CogStim_it_*	0	100	0.403	2.553
*IntStim_it_*	0	50	0.081	0.837
*Status_it_*	0	1	0.032	0.177
*ComRec_it_*	0	258	0.322	2.977
*IdeaPos_it_*	0	583	4.141	16.075
*Tenure_it_*	0	194	23.092	36.757
*WC_it_*	0	514	0.835	5.743
*WPS_it_*	0	131	0.367	2.073
*Sixltr_it_*	0	100	0.764	4.22
*Negate_it_*	0	33.333	0.027	0.359

**Table 4:** Correlation matrix.

**Variables**	**0**	**1**	**2**	**3**	**4**	**5**	**6**	**7**	**8**	**9**	**10**	**11**	**12**	**VIF**
0. *Idea_it_*	1.000													-
1. *EmoApp_it_*	0.423	1.000												3.13
2. *IndCons_it_*	0.287	0.310	1.000											1.36
3. *Costume_r_*	0.419	0.369	0.364	1.000										2.34
4. *IntStim_it_*	0.260	0.212	0.197	0.567	1.000									1.52
5. *Status_it_*	0.515	0.754	0.401	0.489	0.302	1.000								2.89
6. *ComRec_it_*	0.407	0.228	0.242	0.401	0.257	0.369	1.000							1.43
7. *IdeaPos_it_*	0.160	0.039	0.047	0.099	0.064	0.060	0.146	1.000						1.03
8. *Tenure_it_*	0.004	0.000	0.000	0.004	0.005	0.001	0.005	0.020	1.000					1.00
9. *WC_it_*	0.421	0.258	0.356	0.558	0.357	0.379	0.479	0.114	0.002	1.000				3.32
10. *WPS_it_*	0.458	0.409	0.464	0.661	0.417	0.529	0.497	0.108	0.002	0.826	1.000			4.69
11. *Sixltr_it_*	0.448	0.717	0.420	0.516	0.291	0.656	0.368	0.071	0.001	0.482	0.615	1.000		2.89
12. *Negate_it_*	0.207	0.108	0.166	0.390	0.347	0.184	0.243	0.062	0.004	0.341	0.375	0.248	1.000	1.26

### Empirical analysis

Table 5 presents the estimation results of the OLS FE models. Model 1 only includes control variables; Model 2 adds the verbal persuasion variables and the moderator; Models 3, 4, 5, and 6 examine the moderating effects of status by sequentially adding interaction items. To control for the overdispersion, potential autocorrelation and heteroskedasticity, we adopt cluster-robust standard errors (Chen et al., 2019a).

**Table 5:** Regression results.

**Verbal Persuasion dimension**	**Variable**	**Model 1**	**Model 2**	**Model 3**	**Model 4**	**Model 5**	**Model 6**
Emotional feedback	Emotional approval		0.001*** (0.000)	−0.000 (0.000)	−0.000 (0.000)	−0.000 (0.000)	−0.000 (0.000)
	Individualized consideration		0.000 (0.001)	0.000 (0.001)	−0.003 (0.003)	−0.002 (0.003)	−0.002 (0.003)
Constructive feedback	Cognitive stimulation		0.002*** (0.001)	0.002*** (0.001)	0.002*** (0.001)	0.001 (0.002)	0.002 (0.002)
	Intellectual stimulation		0.001 (0.002)	0.001 (0.002)	0.001 (0.002)	0.001 (0.002)	−0.004 (0.003)
Moderator	Status		1.244*** (0.012)	1.244*** (0.012)	1.244*** (0.012)	1.245*** (0.012)	1.245*** (0.012)
Moderating effects	Emotional approval ×Status			0.002*** (0.000)	0.002*** (0.000)	0.002*** (0.000)	0.002*** (0.000)
	Individualized consideration×Status				0.004 (0.003)	0.003 (0.003)	0.003 (0.003)
	Cognitive stimulation×Status					0.002 (0.002)	0.000 (0.002)
	Intellectual stimulation×Status						0.007* (0.004)
Control variables	Comments received	0.005*** (0.001)	0.004*** (0.001)	0.004*** (0.001)	0.004*** (0.001)	0.004*** (0.001)	0.004*** (0.001)
	Ideas posted	0.061*** (0.007)	0.047*** (0.006)	0.047*** (0.006)	0.047*** (0.006)	0.047*** (0.006)	0.047*** (0.006)
	Tenure	−0.004*** (0.001)	−0.004*** (0.001)	−0.004*** (0.001)	−0.004*** (0.001)	−0.004*** (0.001)	−0.004*** (0.001)
	Tenure2	0.000** (0.000)	0.000** (0.000)	0.000** (0.000)	0.000** (0.000)	0.000** (0.000)	0.000** (0.000)
	Average word count	0.001 (0.000)	0.001* (0.000)	0.001* (0.000)	0.001** (0.000)	0.001** (0.000)	0.001** (0.000)
	Words per sentence	−0.003** (0.001)	−0.003** (0.001)	−0.003** (0.001)	−0.003** (0.001)	−0.003** (0.001)	−0.003** (0.001)
	Sixltr	0.001* (0.000)	−0.000 (0.000)	−0.000 (0.000)	−0.000 (0.000)	−0.000 (0.000)	−0.000 (0.000)
	Negate	−0.006** (0.003)	−0.008** (0.003)	−0.007** (0.003)	−0.007** (0.003)	−0.006** (0.003)	−0.005 (0.003)
	Monthly dummies	Yes	Yes	Yes	Yes	Yes	Yes
	Observations	266,033	266,033	266,033	266,033	266,033	266,033
	*R*-squared	0.030	0.287	0.287	0.287	0.287	0.287
	# of users	15,649	15,649	15,649	15,649	15,649	15,649
	*F*	40.17***	450.91***	436.24***	422.81***	411.98***	399.74***

Standard errors are reported in parentheses; *p < 0.1; **p < 0.05; ***p < 0.01.

Based on Model 2, which contains all independent and control variables for time effects, we interpret the main effects of the hypotheses. As shown in Column 4 of Table 5, emotional approval is positively related to idea contribution (value of *p* <0.01), whereas individualized consideration has no significant influence. The results indicate that peers’ emotional support language use, especially emotional approval, plays a crucial role in promoting individuals’ subsequent idea contribution. In contrast, individualized consideration seems unimportant in our research setting. In addition, the cognitive stimulation words used by peers exert a significant positive impact on idea contribution (value of *p* <0.05), which verifies the promoting effect of constructive feedback on individuals’ consequent contribution. However, the intellectual stimulation words have no effect. This is different from the results of prior related work, which studied the intellectual stimulation effect of leaders’ exclusive words on their followers’ creativity (Becker et al., 2021). The reason for this may be due to the different statuses of verbal persuasion providers. Exclusive words represent questions and challenges, indicating a certain critical tone. The questioning from leaders can stimulate followers’ effort, but it does not necessarily apply to peers. In general, we find partial support for both H1 and H2.

For the moderating effects of receiving verbal persuasion from opinion leaders, the last column of Table 5 demonstrates that the interaction term of emotional approval and status is significantly positive (value of *p* <0.01), indicating that emotional support, especially the emotional approval from opinion leaders, has a stronger promoting effect on individuals’ idea contribution. However, consideration from peers does not work, even when it is from opinion leaders. In addition, the interaction term of cognitive stimulation and status is insignificant. The result suggests that constructive feedback that reflects cognitive processes (e.g., suggestion) always facilitates individuals’ idea contribution, regardless of the status of the provider. Interestingly, it was found that the coefficient of intellectual stimulation in Model 2 is not significant, but its interaction term with status is positively significant (value of *p* = 0.055). This reflects that the intellectual stimulation effect of exclusion words is dependent on the status of the provider, corroborating that leaders can use exclusion words to stimulate their followers’ innovation. In conclusion, the results confirm the critical role of the source of verbal persuasion, and Hypotheses H3a and H3b are partially supported.

With regard to the controls, it was shown that the results are basically consistent with existing studies (Chen et al., 2012; Chan et al., 2015; Dong et al., 2020). The number of comments received from peers is positively related to individuals’ continuous contributions. The number of ideas released before, known as experience, can also positively affect users’ continuous contribution. There is an inverted U-shaped relationship between tenure and individuals’ continuous contribution. Previous studies have also produced similar results, which demonstrate that individuals may stop contributing or even leave the community after the initial contribution. Lack of motivation for users to continue to participate is a common problem in the online community. Additionally, many other linguistic styles that have been proven to have an impact on user behavior, such as word count, words per sentence and negating words, have also shown different effects in our model.

### Robustness checks

We evaluate the robustness and consistency of the main effect by utilizing an alternative definition of independent variable, subsample regression, and different estimation methods, as summarized in Table 6. First, we assessed the robustness by changing the measurement of emotional approval. The original measurement only focuses on the positive words in the comments without considering the influence of negative words. To solve the above problem, we apply the *tone* LIWC category to further examine our hypotheses. The *tone* category in LIWC calculates the emotions of sample language with a relative measure between 1 and 100, where numbers above (below) 50 suggest a more positive (negative) emotional tone. Table 6 Column 2 shows that the direction and significance of the coefficient estimates remain the same as in Model 2, indicating that our results are robust to the alternative. In addition, we also evaluate the robustness by randomly selecting 5,000 users for subsample regression. The results are shown in Table 6, Column 3, which yield consistent results derived from our full sample. Finally, we apply both the FE negative binomial model and the Poisson model to conduct the estimation. The results shown in Columns 4 and 5 of Table 6 are found to be still consistent with the main analysis. Thus, the results of several robustness tests did not reveal discrepancies with our primary findings.

**Table 6:** Robustness check of the effects of verbal persuasion on innovation.

	**Posemo**	**Sub-sample**	**FE Nbreg**	**FE Poisson**
**Variable**	**Model 7**	**Model 8**	**Model 9**	**Model 10**
Emotional approval	–	0.001*** (0.000)	0.006*** (0.001)	0.007*** (0.001)
Tone	0.000*** (0.000)	–	–	–
Individualized consideration	−0.001 (0.001)	0.002 (0.002)	0.005 (0.006)	0.007 (0.007)
Cognitive stimulation	0.002** (0.001)	0.004** (0.002)	0.011*** (0.003)	0.010*** (0.003)
Intellectual stimulation	0.000 (0.002)	0.003 (0.004)	0.001 (0.007)	0.002 (0.006)
Comments received	0.005*** (0.001)	0.004** (0.002)	0.006*** (0.001)	0.005*** (0.001)
Ideas posted	0.061*** (0.007)	0.082*** (0.009)	0.041*** (0.002)	0.066*** (0.018)
Tenure	−0.004*** (0.001)	−0.006*** (0.001)	−0.017*** (0.001)	−0.037*** (0.008)
Tenure2	0.000** (0.000)	0.000 (0.000)	0.000*** (0.000)	0.000* (0.000)
Average word count	0.001* (0.000)	0.002* (0.001)	0.004*** (0.001)	0.003** (0.001)
Words per sentence	−0.005*** (0.001)	−0.008*** (0.003)	−0.005 (0.004)	−0.007 (0.005)
Sixltr	−0.001 (0.000)	−0.001 (0.001)	0.001 (0.002)	0.001 (0.002)
Negate	−0.008** (0.003)	−0.011* (0.006)	−0.023 (0.017)	−0.048** (0.019)
Intercept	−0.008 (0.025)	−0.025 (0.036)	−1.552*** (0.034)	
Monthly dummies	Yes	Yes	Yes	Yes
Observations	266,033	85,000	239,819	239,819
# of users	15,649	5,000	14,107	14,107
Log-likelihood	-		−58933.136	−62179.699
Wald *χ*^2^	-		1170.35	886.17

*p < 0.1; **p < 0.05; ***p < 0.01.

## Discussion

This paper focuses on the continuous contribution of members in an open innovation community, an essential prerequisite for ensuring the stable development of the community. Based on the creative self-efficacy theory, we explore the role of verbal persuasion through emotional support and constructive feedback on the continuous idea contribution of individuals in the innovation community, as well as the moderating role of the verbal persuader’s status. By conceptualizing and operationalizing verbal persuasion dimensions, this paper provides new insights into whether and how an OIC peer’s comment can motivate members to continue making contributions.

As theorized, it was found that emotional support, especially emotional approval, has a positive effect on members’ continuous idea contributions, while individualized consideration does not seem to work. This illustrates the importance of recognition and approval in motivating continuous idea contributions from community members. Along with the results of the moderating effect, we can recognize that this type of verbal persuasion has a stronger promoting effect if it comes from peers with a high status, such as opinion leaders in the community. However, we did not find a significant impact of another aspect of emotional support (i.e., concern) on continuous idea contribution, which may be because our research context is different from existing research (Becker et al., 2021), and there is less individualized consideration behavior in the household sector innovation community.

In terms of the stimulating effects of constructive feedback on continuous idea contribution, support from cognitive stimulation is also found, which validates the cognitive benefits of peer feedback in promoting user engagement (Ogink and Dong, 2017). Moreover, we do not find any positive or negative moderating effect of the status of the verbal persuasion provider on the relationship between cognitive stimulation words and continuous contribution, indicating that the stimulation of cognitive process words is not affected by the source. This highlights its importance and universality from the side. In addition, while intellectual stimulation is not significant in the main effect, the interaction term with status is significantly positive, suggesting that such verbal persuasions with questioning and challenging tones only work when the status of the provider is high.

Even if the results are not entirely consistent with our hypothesis, we find that verbal persuasion from peers significantly affects members’ continuous idea contributions in an open innovation community. It represents an important driver of individuals’ subsequent engagement after the initial contribution. Thus, its uses should be guided and designed to take full advantage of the potential boost to user contribution.

### Theoretical implications

This study offers several theoretical implications. First, the findings broaden the literature about the factors that influence user continuous contribution in online communities by detailing how peers’ language use in feedback can facilitate the development of ideas. Therefore, we respond to calls to investigate how the sentiment or other linguistic styles of peer feedback (i.e., comments) impact future contribution behaviors for the focal participant (Chen et al., 2019b). Previous research on peer feedback has predominantly tended to summarize unstructured text with structured proxies, focusing on the superficial characteristics of comments, such as amount (Chen et al., 2012), diversity (Yang and Han, 2019) and timeliness (Chen Q. et al., 2020). However, these quantifiable metrics often overshadow the richness of the text (Berger et al., 2020). Based on our findings, we shed light on how peers’ language use affects members’ continuous idea contributions in OICs. With the prevalence of open innovation, firms increasingly turn to establish OICs, gathering external ideas for their innovation; meanwhile, a growing number of innovation communities for individuals are emerging. Identifying effective strategies to encourage continuous contributions from participants is essential for both practitioners and researchers (Beretta et al., 2018).

Second, this study contributes to the creative self-efficacy theory. Previous research on creative self-efficacy has been performed primarily in organizational management (Liao et al., 2021). This paper extends the application of creative self-efficacy by applying it to an open innovation community. Furthermore, prior research related to sources of self-efficacy mainly focused on direct and vicarious experiences (Riedl and Seidelc, 2018; Liu et al., 2020), while the role of verbal persuasion was underestimated. This paper is among the first to focus on explaining the effects of verbal persuasion, especially the language use of persuasion on creative self-efficacy. By applying well-established creative self-efficacy theory and identifying linguistic cues of verbal persuasion, we bridge peer feedback, verbal persuasion and user innovation research, therefore, affirming that peers can affect other community members by the specific language they use. Meanwhile, we enrich and refine the study of verbal persuasion by dividing the type (e.g., emotional support and constructive feedback) and segmenting the dimensions. In particular, we show that verbal persuasions are not universally effective. For example, while peers’ emotional approval exerts positive effects on individuals’ contributions, consideration from peers does not work. In addition, in contrast from emotional approval, the effect of constructive feedback, which reflects cognitive processes, is not influenced by the source. These results reveal the differential influences that emerge within and across the verbal persuasion dimensions, indicating that studies on peer feedback should be conducted based on language dimensions instead of valuing each form of feedback equally.

Third, this study notably introduces the status of a verbal persuasion provider as a moderating variable. In previous studies, scholars were usually interested in the status of the focal user, investigating its motivating role in promoting user engagement (Goes et al., 2016; Dong et al., 2020). The current work focuses on the interacting effect of the status of peers and verbal persuasion. The findings disclose that provider’s status exerts differential moderating effects among different dimensions of verbal persuasion. For example, emotional approval from opinion leaders can exert stronger effects on individuals’ continuous contribution; opinion leaders can stimulate individuals intellectually through a questioning or challenging tone, while it is difficult for ordinary peers to make a difference. This finding is in accordance with the prior work that highlights that when a person with experience, referred to as the model, offers verbal persuasion, the recipient’s efficacy beliefs should be strengthened more (Mellor et al., 2006). The undertaking of this study extends our knowledge about how the source of verbal persuasion influences its effects in OICs. Overall, this research sheds light on how provider’s status interacts with verbal persuasion in affecting individuals’ continuous idea contributions.

### Managerial implications

This study has insights for open innovation community management. A practical issue for OIC managers is how to motivate continuous engagement and contribution from members, considering that the long-term success of these communities is heavily dependent on members’ sustained contributions (Chen et al., 2019b). In this study, we highlight the relevance of language use for peer feedback. According to our results, it can increase members’ idea contributions, thus, providing a new perspective on how to facilitate members’ continuous contributions. The community can encourage peer-to-peer communication and commenting activities through incentives (e.g., badge mechanism, homepage exhibition) and provide some guidance on the commenting language according to research results, thereby motivating new entrants and stimulating existing ones to make further idea contributions.

Prior work has emphasized that social interaction, especially textual comments, can positively affect users’ subsequent engagement and contribution (Chen et al., 2019b). Our research also confirms the importance of verbal persuasion from peers. However, there are still many ideas in the community that do not receive any responses for various reasons, resulting in a newcomer leaving the community at that point. Drawing on chatbots in e-commerce, which are used widely to communicate with customers (Araujo, 2018), we suggest building an automated comment bot. Referring to research on language style, training comment-bots to post comments with specific language has potential stimulant effects on members’ continuous idea contribution behavior.

Our results reveal that the status of verbal persuasion providers matters. However, in practice, the recipient may not be aware of the commenter’s status without clicking and transferring to the commenter’s homepage, especially if there are many commenters. Therefore, we suggest that the commenting interface of ideas in the community could be changed accordingly. The current design allows the focal recipient to obtain the avatar and nickname of the commenter, which is not useful for unknown commenters. Thus, it is recommended to add representations that reflect the identity and status of the commenter, which would take full advantage of our findings to maximally promote members’ continuous contributions.

### Limitations and future directions

There are several limitations in the current study. First, we mainly center around the objective data collected from a single household sector innovation community, which may limit the generalizability of the results. As shown in prior research, various types of online communities, such as open source software communities (Piezunka and Dahlander, 2019), Q&A communities (Jin et al., 2015) and online health care communities (Peng et al., 2020) all provide peer feedback mechanisms, and it remains to be verified whether the conclusions of this paper are applicable to the above communities. Thus, much broader context-oriented studies on verbal persuasion from peers are required for further explorations.

Second, this study focuses on only one aspect of continuous contributions, i.e., the quantity of ideas, neglecting the impact on the quality of ideas. However, the quality of contributions is also a crucial prerequisite for community development and success (Chen et al., 2012). Moreover, the same verbal persuasion may exert differential, or even contradictory, effects on contribution quantity and quality (Becker et al., 2021). Future studies, therefore, may investigate the relationship between verbal persuasion from peers and the quality of continuous contribution.

Third, although LIWC’s text analysis methods are popular due to their ease of use and academic validity (Tausczik and Pennebaker, 2010; Piezunka and Dahlander, 2019), dictionary-based text analytical approaches cannot take the contextual background into consideration. Thus, verbal persuasion-related studies might benefit from large-scale text analyses that rely on machine learning, topic modeling algorithms or human coding to explore the impact of peers’ textual content on recipients’ subsequent behaviors.

## Data availability statement

The raw data supporting the conclusions of this article will be made available by the authors, without undue reservation.

## Author contributions

JZ contributed to all the phases of the study from conception and design of the study, statistical analysis and results interpretation. GQ contributed to conception of the study and polish the manuscript. CS contributed to theoretical literature review and data collection. JC contributed to supervision and the revision of the work. All authors contributed to the article and approved the submitted version.

## Funding

This research was partially supported in the collection, analysis, and interpretation of data by the National Natural Science Foundation of China under Grant No. 72072103, and the Natural Science Foundation of Shandong Province under Grant No. ZR2021QG014.

## Conflict of interest

The authors declare that the research was conducted in the absence of any commercial or financial relationships that could be construed as a potential conflict of interest.

## Publisher’s note

All claims expressed in this article are solely those of the authors and do not necessarily represent those of their affiliated organizations, or those of the publisher, the editors and the reviewers. Any product that may be evaluated in this article, or claim that may be made by its manufacturer, is not guaranteed or endorsed by the publisher.
